# Methcathinone Neurotoxicity in the Rat Prefrontal Cortex by Integrated Synaptic Changes and Transcriptome Analysis

**DOI:** 10.1111/adb.70113

**Published:** 2025-12-18

**Authors:** Rukui Zhou, Yingwen Xu, Chunming Xu, zhe Chen, Jieping Lv, Keming Yun, Zhiwen Wei

**Affiliations:** ^1^ School of Forensic Medicine Shanxi Medical University Jinzhong Shanxi China; ^2^ School of Basic Medical Sciences Shanxi University of Chinese Medicine Jinzhong Shanxi China; ^3^ Department of Anesthesiology The First Hospital of Shanxi Medical University Taiyuan Shanxi China

**Keywords:** methcathinone, neurotoxicity, synapse, transcriptomics

## Abstract

Long‐term abuse of methcathinone reduces grey matter volume in the prefrontal cortex and consequently impairs learning and memory abilities. However, the exact mechanism of damage remains unknown. Therefore, this study aimed to analyse the potential mechanisms underlying methcathinone‐induced neural damage using transcriptomic analysis. Accordingly, 32 Sprague Dawley rats were randomly divided into four groups: control, low‐dose, medium‐dose and high‐dose. Low, medium and high methcathinone doses (0.25, 5 and 20 mg/kg) were administered to the animals in the three treatment groups once daily via intraperitoneal injection for 2 weeks. Finally, the learning and memory functions of all the animals were tested using the Morris water maze. Electron microscopy and Golgi staining were used to observe changes in synaptic structure, and transcriptome sequencing was performed in the prefrontal cortex of the control and high‐dose groups. Key differentially expressed genes were quantified using quantitative real‐time reverse transcription polymerase chain reaction. Collectively, methcathinone induced learning and memory decline in rats and destroyed the synaptic structure of the rat prefrontal cortex. In the transcriptomic analysis, 1457 (694 up‐regulated and 763 down‐regulated) genes were differentially expressed in the prefrontal cortex of rats in the high‐concentration group compared to that in the control group. Gene Ontology terms and Kyoto Encyclopedia of Genes and Genomes analysis revealed that differential genes were enriched in synapses, neurotransmitter systems, homeostasis of Ca^2+^ concentration and membrane potential regulation. This indicates that methcathinone adversely affects neurotransmitter regulation, Ca^2+^ signalling and membrane potential regulation, thereby destroying synapse structure and causing learning and memory dysfunction. Combined with the above molecular mechanisms, seven key genes were identified: nerve growth factor (NGF), dopamine receptor D1 (DRD1), dopamine receptor D2 (DRD2), solute carrier family 1 member 2 (SLC1A2), calcium/calmodulin‐dependent protein kinase II alpha (CAMK2A), synaptotagmin 1 (SYT1) and glutamate ionotropic receptor *N*‐methyl‐d‐aspartate type subunit 2A (GRIN2A). This study demonstrates that methcathinone causes neural damage and provides possible molecular mechanisms and target genes to clarify the mechanism of methcathinone‐induced neural damage.

## Graphical abstract

Integrating behavioural, ultrastructural and transcriptomic analyses, this study reveals that subchronic methcathinone exposure damages prefrontal synaptic architecture and alters neuroplasticity‐related gene networks, providing mechanistic insight into its neurotoxic and cognition‐impairing effects.

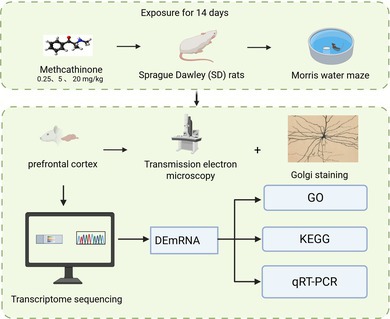

## 1 Introduction

The abuse of methamphetamine and other stimulants constitutes a major global public health problem. In recent years, a novel synthetic cathinone, methcathinone (MCAT), commonly known as the “zombie drug”, has emerged as a dangerous and widely abused alternative [1, 2]. The drug is a β‐keto psychostimulant in the amphetamine class and has a similar structure to methamphetamine [3, 4]. Favoured for its heightened potency, low cost and availability, MCAT abuse has been linked to an increase in cases of addiction, serious neuropsychiatric manifestations and mortality, thereby presenting an emergent threat to public health [5, 6, 7].

Existing research has preliminarily elucidated the neurotoxicity of MCAT [8, 9, 10]. As a substrate for dopamine (DA) and serotonin (5‐HT) transporters, MCAT induces a massive, non‐physiological release of these neurotransmitters and disrupts vesicular storage. This not only produces intense euphoria and hallucinations but also leads to a pronounced reduction in tissue DA content and DAergic terminal protein markers, including DA transporter and tyrosine hydroxylase synthase [11, 12, 13]. Furthermore, MCAT generates an increase in reactive oxygen (ROS) and nitrogen species, which damage cellular components. ROS are produced during MCAT metabolism, leading to neuronal lipid peroxidation and DNA damage [14, 15]. Long‐term MCAT administration markedly reduces the levels of superoxide dismutase and glutathione in the brain tissue of mice, thereby indicating an imbalance in the antioxidant system [16, 17]. Simultaneously, mitochondrial respiratory chain complex activity is inhibited, adenosine triphosphate production is reduced, and changes in mitochondrial membrane permeability trigger calcium overload, thereby activating apoptotic pathways, such as caspase‐3 [18, 19]. MCAT induces microglia to release proinflammatory factors, such as tumour necrosis factor‐α and interleukin‐6, by activating the Toll‐like receptor 4/nuclear factor kappa B signalling pathway, thereby amplifying neuroinflammation [20, 21]. However, the key triggering mechanism for this series of damage remains unclear. Simultaneously, MCAT is manufactured through an illicit process that involves oxidising ephedrine and pseudoephedrine contained in readily available drugs with potassium permanganate [22]. Intravenous administration of such MCAT preparations exposes users to high Mn loads. Mn accumulation may result in encephalopathy and trigger secondary pathogenic mechanisms, such as mitochondrial and autophagy–lysosomal pathway dysfunction [23, 24].

The prefrontal cortex (PFC) is a key site in learning and memory [25]. Clinical observations have revealed reduced grey matter in the PFC and cognitive impairment in long‐term MCAT abusers [26]. Changes in gene expression in related brain circuits alter the structural morphology of synapses, which ultimately adjust learning and memory [27, 28, 29]. The key genes, pathways and molecular events through which MCAT induces synaptic remodelling and cognitive dysfunction in the PFC remain to be elucidated. To clarify the mechanism and process of MCAT‐induced neural damage, the effects of MCAT on the learning and memory functions of rats were examined, and changes in synaptic morphology were monitored. Combined with high‐throughput sequencing, the transcriptome differences of the PFC of the blank control group and the high‐concentration MCAT group were compared, and the related functions and enriched pathways of differentially expressed genes (DEGs) were analysed to identify related biological processes and pathways. This approach aims to establish an integrated pathophysiological model that connects molecular alterations to the behavioural manifestations of cognitive deficits, thereby improving understanding of the neurotoxic mechanism of MCAT.

## 2 Materials and Methods

### 2.1 MCAT Exposure Rat Model

Eight‐week‐old male Sprague–Dawley rats (*n* = 32) were housed under standard conditions (22°C ± 2°C, 45%–55% humidity, noise < 60 dB and 12‐h light–dark cycle), with free access to clean water and standard food. The rats were randomly divided into four groups (*n* = 8 rats per group): control, low MCAT (0.25 mg/kg MACT), medium MCAT (5 mg/kg MACT) and high MCAT (20 mg/kg MACT). After 1 week of adaptive feeding, the rats were administered MCAT via intraperitoneal injection daily for 2 weeks. All animal experiments were approved by the Institutional Animal Care and Use Committee of Shanxi Medical University (2021‐338).

### 2.2 Morris Water Maze

After constructing the rat model, the Morris water maze (MWM) was used to evaluate the learning and memory of all rats. The maze was a circular pool 60 cm in height and 130 cm in diameter. The pool was divided into four quadrants, and the platform was placed in the middle of the northeastern quadrant and submerged 2 cm below the water surface. The day before the test, each rat was allowed to swim for 120 s to adapt to the environment. Five days before the formal test, a positioning navigation experiment was conducted. The rats entered the pool from the four directions S, W, NE and SE in different orders. If the rat found the platform and remained stable for 10 s, the system was stopped, and the escape latency of the rat was recorded. If the rat did not find the platform within 120 s, it was manually guided to the platform and allowed to remain for 10 s. The daily escape latency of the rats was the average of four directions on that day. On the sixth day, the spatial exploration experiment was conducted. The platform was removed, and the rats entered the pool from the SW quadrant. The system automatically recorded the number of times the rat crossed the platform and the time it remained in the NE quadrant within 120 s. A camera placed above the maze was used to record the swimming path of each rat, and the data were analyzed using Smart v3.0 software.

### 2.3 Transmission Electron Microscopy

Transmission electron microscopy (TEM) was used to observe synaptic structures of neurons in the PFC. After rats were anaesthetised and perfused with 4% paraformaldehyde, their brains were quickly removed, and the PFC was isolated. The PFC was cut into 1‐mm^3^ tissue slices and fixed in 2% glutaraldehyde at 4°C for 2 h. After washing the tissue blocks four times with a buffer solution, the slices were fixed in 1% OSO 4 for 90 min and dehydrated with acetone gradients for 15 min at each gradient. The tissue was embedded in epoxy resin 618 and cut into 50‐nm sections using an ultramicrotome (LKB, Norrbotten, Sweden). Thereafter, sections were stained with uranyl acetate and lead citrate for 30 min and then observed using TEM (JEM‐100CXII, Japan). The thicknesses of the synaptic cleft and postsynaptic density regions were calculated using ImageJ software.

### 2.4 Golgi Staining and Counting

The procedure was performed according to the instructions of the FD Fast Golgi Staining Kit (FD Neuro Technologies, Columbia, MD, USA). The prefrontal cortices of the rats were collected and immersed in a mixture of solutions A and B, which were prepared 24 h in advance. The mixture was changed the next day and stored in the dark at room temperature for 2 weeks. Brain tissue was removed, immersed in Solution C and stored in the dark for 24 h. Subsequently, the solution was changed for another 4 days. The tissue was removed and cooled into blocks in pre‐cooled isopentane, and surface isopentane was removed. The tissue was cut into 100‐μm slices using a cryostat microtome (Leica, Wetzlar, Hessen, Germany) and attached to a slide. The slides were then placed in a mixture of Solutions D and E. After the reaction, slides were washed with distilled water and dehydrated in graded ethanol for 60 s. The tissue was cleared with xylene solution for 2 min. Finally, the discs were sealed using neutral resin, and the images were observed under a microscope (Nikon Corporation, Minato‐ku, Tokyo, Japan). The density and length of dendritic spines were analysed using ImageJ software (National Institutes of Health, Bethesda, MD, USA).

### 2.5 RNA Extraction, Library Construction and Sequencing

RNA sequencing (RNA‐seq) was performed on six rats; three from the control group and three from the 20 mg/kg MACT‐treated rat model. Total RNA was extracted from the entire PFC according to the manufacturer's instructions. Agarose gel electrophoresis was performed to detect RNA degradation and contamination. RNA purity was measured using an Eppendorf μCuvette G1.0 (Eppendorf, Hamburg, Germany); RNA integrity was assessed using an Agilent 2100 Bioanalyzer system (Agilent Technologies, Santa Clara, CA, USA), and RNA concentration was detected using the Qubit RNA Assay Kit in a Qubit 2.0 Fluorometer (Life Technologies, Carlsbad, CA, USA). The RNA concentration could not be < 400 ng/μL.

After quality inspection, 3 μg of RNA was used for complementary DNA (cDNA) library construction for each sample. First, ribosomal RNA (rRNA) was removed using the Epicentre Ribo‐Zero rRNA Removal Kit (Illumina, San Diego, CA, USA), and cDNA libraries were constructed using the VAHTS Universal V8 RNA‐seq Library Prep Kit for Illumina (VAZYME, Nanjing, China). After quality assessment using an Agilent 2100 Bioanalyzer system, cDNA libraries were sequenced on an Illumina HiSeq 2500 platform (Illumina). The differential analysis mainly consists of three steps: (1) First, the original read count is normalised, mainly to correct the sequencing depth; (2) the hypothesis test probability (*p* value) is calculated using a statistical model; (3) multiple hypothesis test correction (BH) is performed to obtain the *p*_adj value (false detection rate). Gene expression and differential expression analysis that yielded log2 fold change (|log2FC|) ≥ 1 and *p*_adj < 0.05 were defined as DEGs.

### 2.6 Gene Ontology Annotation, Kyoto Encyclopedia of Genes and Genomes Pathway Analysis and Protein–Protein Interaction Network of DEGs

Gene Ontology (GO) is a standard term used to annotate biological functions. Kyoto Encyclopedia of Genes and Genomes (KEGG) is a database that collects resources, such as genomes, biological processes, diseases and compounds. GO annotation and KEGG pathway enrichment analysis were performed using a cluster analyser. Protein–protein interaction (PPI) networks are useful for studying the molecular mechanisms of diseases and identifying new drug targets. PPI analysis was performed using the STRING database (https://string‐db.org/), PPI pairs were extracted with a combined score of > 0.4, and the results were calculated with their automatically cited parameters. Subsequently, the PPI network was visualised using the CytoNCA plug‐in in Cytoscape software (Version 3.9.1; https://www.cytoscape.org/) to calculate the betweenness of each protein node.

### 2.7 Real‐Time Quantitative Polymerase Chain Reaction Validation

The RNA‐seq results were validated using real‐time quantitative polymerase chain reaction (RT‐qPCR). Total RNA was extracted from the rat PFC using the HiScript 1st Strand cDNA Synthesis Kit (BYT430, Tiangen, Beijing, China) according to the manufacturer's instructions, and the purity and concentration of RNA were measured using an Eppendorf μCuvette G1.0. RT‐qPCR was performed using AceQQPCR SYBR Green Master Mix (Q121‐02, Jizhenbio, Shanghai, China) in an Applied Biosystems 7500 system (ABI, Waltham, MA, USA). The 20‐μL PCR reaction volume contained 6.4 μL of H_2_O, 0.8 μL of F primer, 0.8 μL of R primer, 2 μL of cDNA and 10 μL of SYBR green mix. The PCR reaction conditions were as follows: 95°C for 10 min, one cycle; 95°C for 10 s, 60°C for 20 s, 72°C for 10 s, 40 cycles. Glyceraldehyde‐3‐phosphate dehydrogenase was used as the mRNA control. Relative RNA expression levels were calculated using the 2^−ΔΔCt^ method.

### 2.8 Statistical Analysis

All data are presented as mean ± standard deviation. Repeated‐measures data were analysed using repeated‐measures analysis. One‐way analysis of variance (ANOVA) was used to compare the results among multiple groups, followed by the least significant difference test when the variances were homogeneous. GraphPad Prism 8 (GraphPad Software Inc., La Jolla, CA, USA) was used to plot the statistical results, and *p* < 0.05 was considered statistically significant.

## 3 Results

### 3.1 MCAT Impaired the Learning and Memory Functions of Rats

**FIGURE 1:**
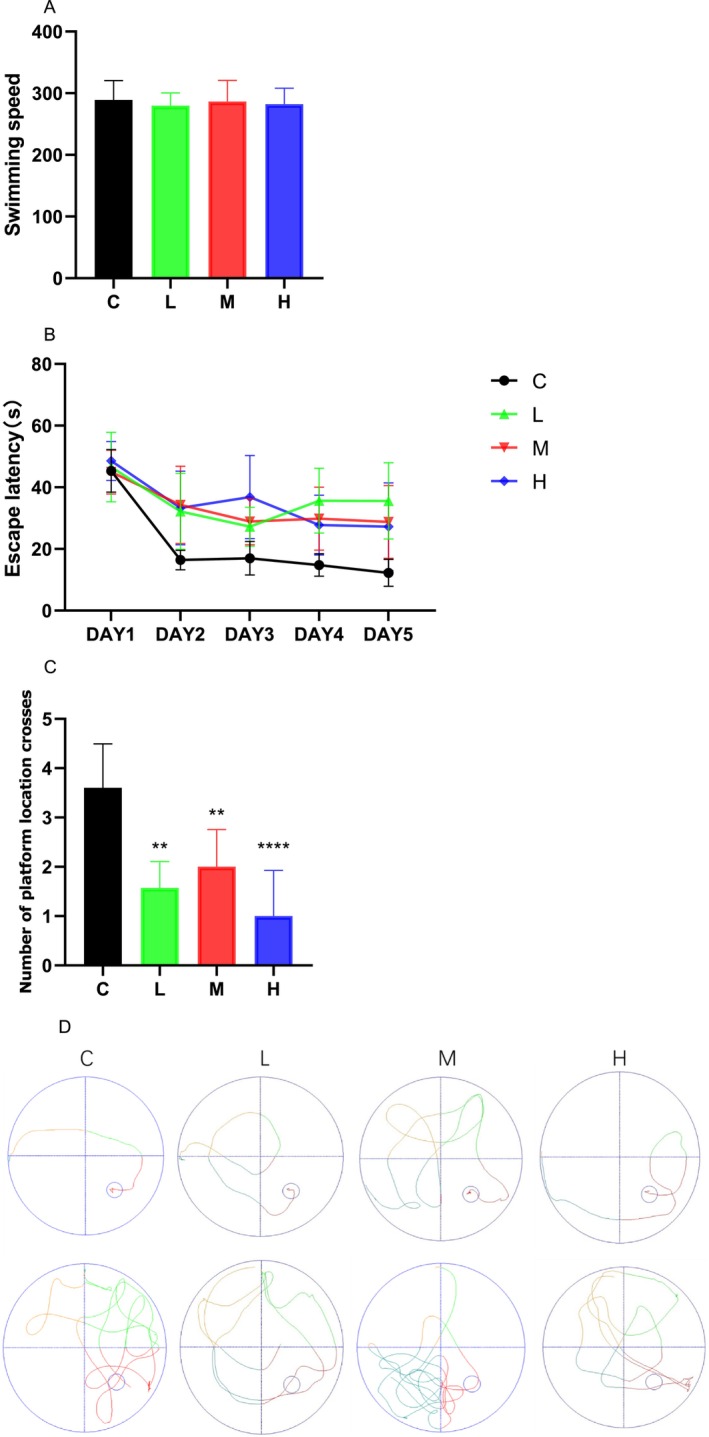
(A) Swimming speed of rats in each group. (B) Escape latency in the navigation test. (C) Number of platform crossings in the spatial exploration test. (D) Representative swimming trajectories of each group on Days 5 and 6. **p < 0.01 and ****p < 0.0001 compared with the control group, *N* = 8. Groups: C (control), L (low‐dose), M (medium‐dose), H (high‐dose). This labeling convention is maintained in all following figures.

Figure 1A shows no statistically significant difference in the swimming speed of each group of rats, indicating that MCAT exposure did not impair their motor ability. The positioning navigation experiment showed that the escape latency of the rats in each group gradually shortened as the training time increased (Figure 1B). One‐way ANOVA was used to analyse the daily escape latency and revealed no statistically significant difference in escape latency between the groups on the first day. The escape latency of rats in the low‐, medium‐ and high‐MCAT groups was significantly prolonged on Days 2–5 (*p* < 0.05 vs. control group), whereas no statistically significant differences were found between the low‐, medium‐ and high‐MCAT groups. In the spatial exploration experiment on the sixth day, the number of times the rats in the low‐, medium‐ and high‐MCAT groups crossed the platform significantly decreased (*p* < 0.05 vs. control group) (Figure 1C). Figure 1D shows the swimming trajectories. As the number of training days increased, the swimming trajectories of rats in the control group became clearer and more purposeful and rats ultimately reached the platform. Although the rats in the low‐, medium‐ and high‐MCAT groups eventually reached the platform, their swimming trajectories were chaotic and random. The results of the water maze test showed that low, medium and high MCAT concentrations impaired learning and memory in rats.

### 3.2 Ultrastructural Damage of Neurons in the Prefrontal Cortex of MCAT‐Exposed Rats

**FIGURE 2:**
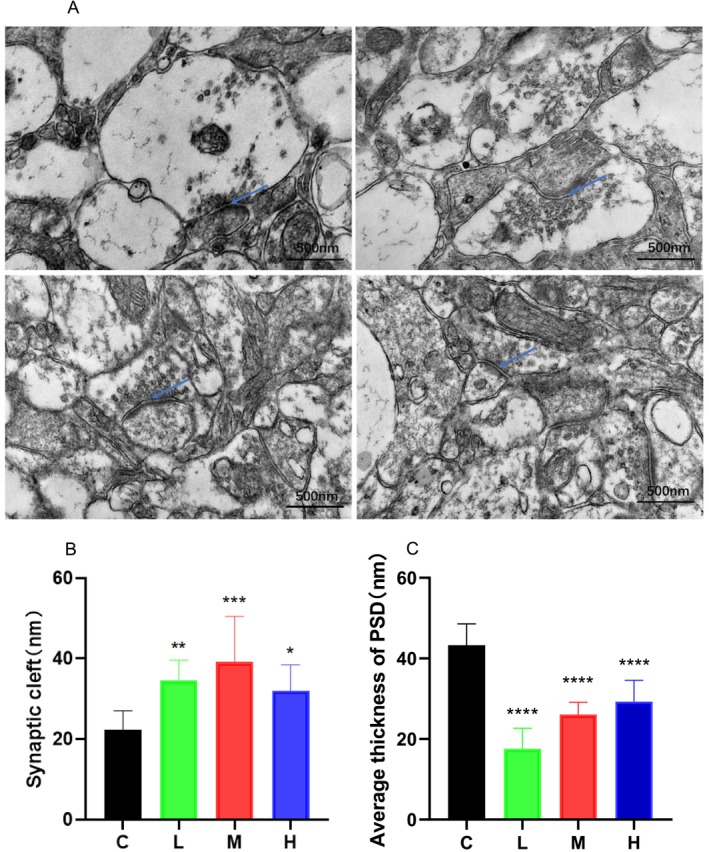
(A) Transmission electron microscopy (TEM) results of rat prefrontal cortex for control, low‐, medium‐ and high‐concentration groups. Magnification = ×60,000. Blue arrows: synaptic structure. (B) Quantification of average synaptic cleft width, *N* = 3. (C) Quantification of average postsynaptic density (PSD) thickness, N = 3. *p < 0.05, **p < 0.01, ***p < 0.001 and ****p < 0.0001 compared with the control group.

The synaptic ultrastructure is the basis of structural synaptic plasticity, and synapses are essential for establishing recognition memory. The synaptic structures observed via electron microscopy are shown in Figure 2A. TEM was used to quantify and compare the width of the synaptic cleft and the thickness of the postsynaptic density (Figure 2B,C). The synaptic morphology of neurons in the control group was good; the synaptic cleft was within 20 nm, and the postsynaptic density was thicker, thus suggesting good synaptic function. However, the synaptic cleft widened, and the postsynaptic density decreased upon low, medium, and high MCAT exposure; hence, synaptic function may have been affected accordingly. Therefore, MCAT exposure may impair synaptic plasticity in PFC neurons.

### 3.3 Effect of MCAT on the Density of Dendritic Spines in the Rat Prefrontal Cortex

**FIGURE 3:**
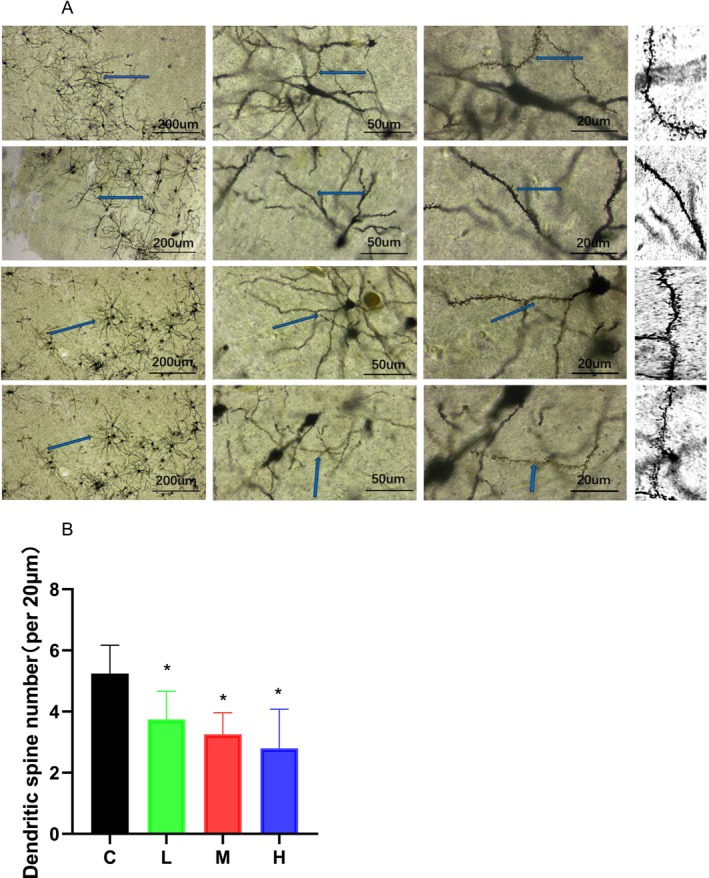
(A) Images of Golgi–Cox–stained neuron and synapse in each group. (B) Dendritic spine number of neurons per 20 μm in the prefrontal cortex of rats in each group, *N* = 3. *p < 0.05 compared with the control group.

Dendritic spines are the original sites of excitatory synaptic transmission in neurons, and their morphology and structure are dynamic, both under normal conditions in vivo and under conditions of synaptic plasticity. Dendritic spine density is related to learning and memory. Figure 3A shows the Golgi–Cox–stained neurons and synapses in each group. The results of Golgi staining in the rat PFC and the quantitative statistics of dendritic spine structure in the rat PFC are shown in Figure 3B. The density of the dendritic spines of neurons in the PFC of the low‐, medium‐ and high‐MCAT groups was significantly lower than that of the control group (*p* < 0.05). In addition, no differences were observed between the low‐, medium‐ and high‐MCAT groups. This indicates that the density of the dendritic spines of neurons in the rat PFC decreased after MCAT exposure.

### 3.4 Sprague–Dawley Rat Prefrontal Cortex Transcriptome

**FIGURE 4:**
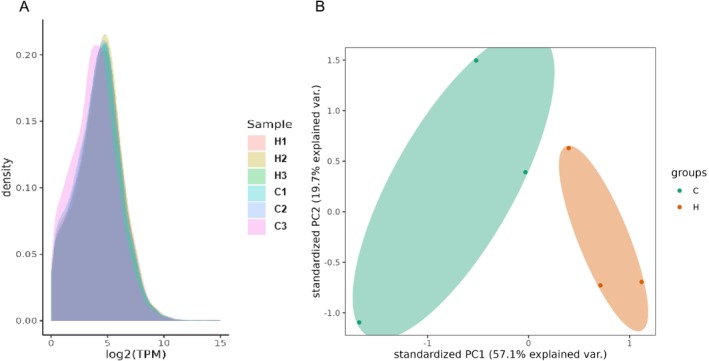
(A) Expression density distribution. The horizontal axis represents log2 (TPM), and the vertical axis represents the gene density, which is the number of genes corresponding to the expression level on the horizontal axis divided by the total number of genes detected to be expressed. Each colour in the graph represents a sample, and the sum of all probabilities is 1. The peak of the density curve represents the region with the highest concentration of gene expression in the entire sample. (B) PCA analysis of mRNA. The values in the axis labels represent the percentage of population variance explained by the corresponding principal component. Points represent each sample, and the same colour and shape represent the same sample group.

For mRNA sequencing, the PFC transcriptome of Sprague–Dawley rats in both the control and MCAT‐treated groups yielded a total of 74.176 GB of clean data, exceeding 11.22 GB per sample. The Q30 base percentage is above 90%, and the GC% content is greater than 46% in all samples. The clean reads from each sample are aligned to a designated reference genome, and the average alignment rate for each sample exceeded 95.82%. Sequencing data are shown in Table 1. Expression density maps (Figure 4A) are plotted based on the log10 (FPKM) values of different samples after processing. The results show that the expression density maps of each sample conformed to a normal distribution, the expression trends of biological replicates are consistent, mRNA expression is relatively stable, and the sequencing data quality is good in each group. Subsequent PCA analysis of the samples using FPKM values reveals significant differences between the groups (Figure 4B).

**TABLE 1:** Statistics of raw data and quality control data.

Sample	Raw bases	Clean reads	Clean bases	Q20%	Q30%	GC%	Average length
C1	11,957,631,000	77,564,698	11,494,053,404	97.1174	91.6237	46.6877	148.1867
C2	13,884,863,400	89,842,044	13,305,426,177	96.9567	91.2564	47.1214	148.098
C3	10,227,531,300	66,311,720	9,816,563,740	96.8942	91.0487	45.6105	148.0366
H1	13,979,900,100	90,624,950	13,427,252,938	97.0381	91.4326	47.4193	148.1629
H2	12,735,754,500	82,632,782	12,228,113,188	97.0873	91.5386	47.3272	147.9814
H3	11,390,030,700	73,864,840	10,963,446,457	97.0406	91.4206	47.5641	148.4258

**Abbreviations:** Average length, average length of reads after quality control; Clean bases, number of bases in the data after quality control; Clean reads, number of sequences in the data after quality control; GC, GC content in the data after quality control; Q20, percentage of bases with a quality value of 20 or higher in the data after quality control; Q30, percentage of bases with a quality value of 30 or higher in the data after quality control; Raw bases, number of raw data bases; Raw reads, number of raw data sequences; Sample, sample name.

### 3.5 Differential Expression of mRNAs Under MCAT Exposure

**FIGURE 5:**
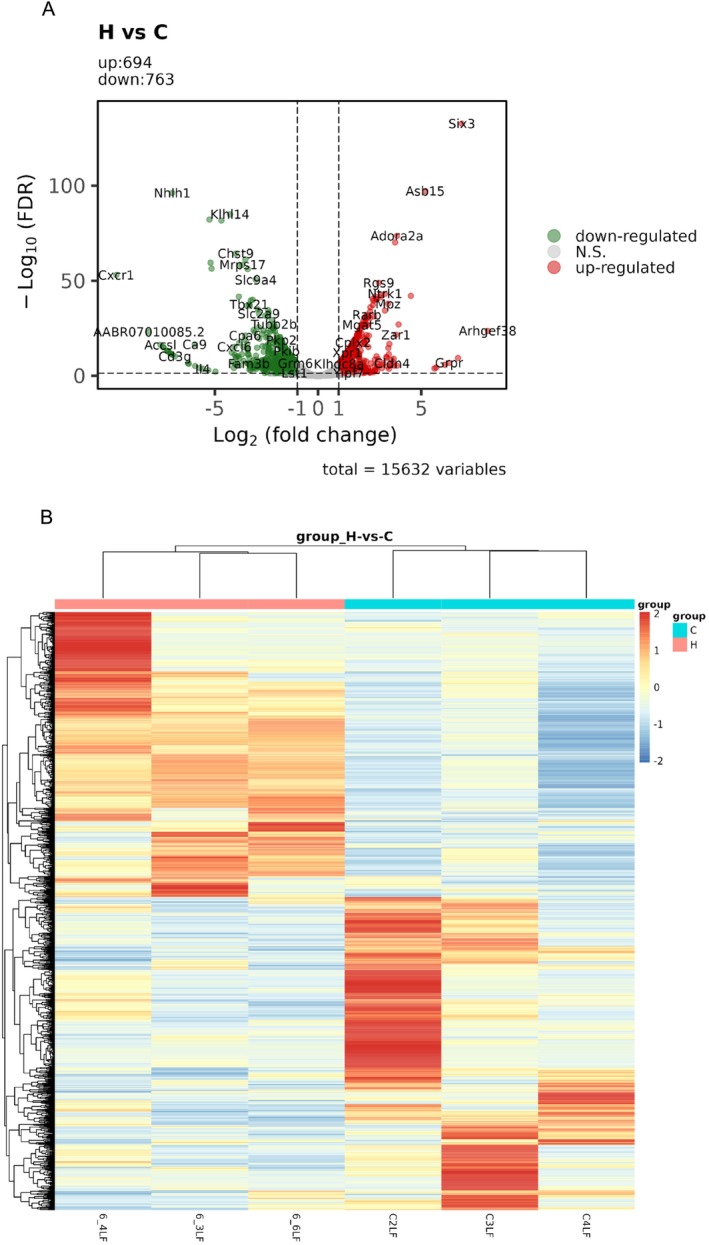
(A) Volcano plot of differentially expressed messenger RNA (DEmRNA). Grey dots represent genes with relatively unchanged expression, green dots represent down‐regulated genes, and red dots represent up‐regulated genes. (B) Hierarchical clustering and heat map analysis of DEmRNAs. Each column in the figure represents a sample, and each row represents a gene. Red represents a higher gene expression in the sample, and green represents a lower gene expression.

To explore the potential biological functions of mRNA in MCAT‐induced neurotoxicity, the mRNA expression patterns in the control and high‐MCAT treatment groups were analyzed. A total of 16,746 mRNAs were identified. The expression profiles were organised into heat maps to display the RNA expression patterns. Values of|log2FC| ≥ 1 and *p*_adj < 0.05 were used as strict criteria for identifying differentially expressed mRNA (DEmRNA). Overall, 1457 (694 up‐regulated and 763 down‐regulated) genes were differentially expressed in the PFC of rats exposed to MCAT compared to those in the control group. Gene expression in the two groups was analyzed using volcano plots (Figure 5A) and heat maps (Figure 5B) and visualised using hierarchical clustering.

### 3.6 Functional Enrichment Analysis of DEGs

**FIGURE 6:**
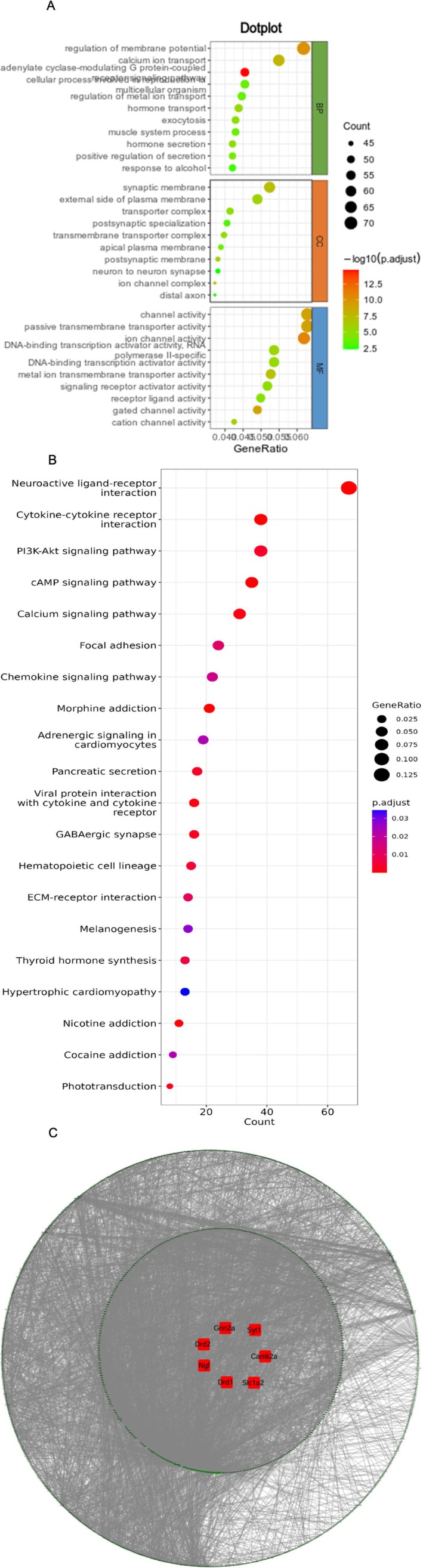
(A) Gene Ontology (GO) analysis of all differentially expressed genes (DEG) between the control group and the high‐methcathinone group. (B) Pathway enrichment statistics of all DEGs between the control group and the high‐methcathinone group. (C) Protein–protein interaction (PPI) plot visualisation using Cytoscape.

To explore the expression of DEGs in the PFC, GO (Figure 6A) and KEGG signalling pathway enrichment analyses were performed (Figure 6B). Collectively, 165 GO terms (adjusted *p* < 0.01) were significantly enriched. In the GO term functional annotation, 104 GO terms were for biological processes (BP), 19 GO terms for cellular components (CC) and 42 GO terms for molecular functions (MF), accounting for 64.03%, 11.51% and 25.45%, respectively. The main enriched subcategories in BP were “regulation of membrane potential,” “calcium ion transport” and “adenylate cyclase‐modulating G protein–coupled receptor signalling pathway.” Moreover, 13 terms related to Ca^2+^, nine terms related to synapses, and five terms related to neurotransmitter regulation were found. The main categories in the MF category were “channel activity,” “passive transmembrane transporter activity” and “ion channel activity,” among which 16 terms related to channel activity were found and accounted for 41.14% of the DEGs in this category. In the CC category, “synaptic membrane,” “external side of plasma membrane” and “transporter complex” were the main enriched subcategories, and 12 terms were related to synapses. These results indicate that MCAT may affect the levels of major neurotransmitters, change the activity of membrane calcium ion channels, affect Ca^2+^ stability and destroy the membrane potential balance, thereby causing damage to synapses in the PFC and subsequent learning and memory dysfunction. The KEGG annotations of DEGs revealed different pathways, and the significantly enriched pathways included neuroactive ligand–receptor interaction, cytokine–cytokine receptor interaction, phosphatidylinositol 3‐kinase (PI3K)–protein kinase B (Akt) signalling pathway, cyclic adenosine monophosphate (cAMP) signalling pathway, calcium signalling pathway, mitogen‐activated protein kinase (MAPK) signalling pathway, pathways of neurodegeneration, multiple diseases, focal adhesion, morphine addiction, amyotrophic lateral sclerosis, Alzheimer's disease and gamma‐aminobutyric acid (GABA)ergic synapses. These pathways often lead to cognitive impairments and are associated with neurodegenerative disease development. The STRING database was used to construct all DEGs in the control and high‐MCAT groups. A PPI network of DEmRNAs was constructed to detect the connections between these DEmRNAs. The PPI network was visualised using Cytoscape, and subnetworks were constructed using the CytoNCA plug‐in of Cytoscape to identify hub genes in the PPI network (Figure 6C). The DEGs involved in dysregulation of neurotransmitter systems, dysfunction in calcium signalling and membrane potential regulation were crossed with the hub genes to obtain key genes, including nerve growth factor (NGF), DA receptor D1 (DRD1), DA receptor D2 (DRD2), solute carrier family 1 member 2 (SLC1A2), calcium/calmodulin‐dependent protein kinase II alpha (CAMK2A), synaptotagmin 1 (SYT1), glutamate ionotropic receptor *N*‐methyl‐d‐aspartate (NMDA) type subunit 2A (GRIN2A) and insulin‐like growth factor 2 (IGF2). The information of key genes is shown in Table 2.

**TABLE 2:** Information on the seven key genes.

Gene ID	Symbol	logFC	*p*	*p*_adjusted	Change
ENSRNOG00000016571	NGF	−1.087552079	2.49277E−08	4.71184E−07	Down
ENSRNOG00000008428	DRD2	3.730208969	3.59617E−74	7.02692E−71	Up
ENSRNOG00000006426	SYT1	1.403171121	1.128E−14	6.25279E−13	Up
ENSRNOG00000005479	SLC1A2	1.656462519	1.74533E−19	1.79494E−17	Up
ENSRNOG00000018712	CAMK2A	1.400262464	1.25113E−14	6.8492E−13	Up
ENSRNOG00000033942	GRIN2A	1.225445001744	1.821E−11	6.01917E−10	Up
ENSRNOG00000023688	DRD1	2.647533928	5.21231E−44	3.25915E−41	Up

*Note:* Hub genes were used to obtain seven key genes: *NGF*, *DRD1*, *DRD2*, *SLC1A2*, *CAMK2A*, *SYT1*, and *GRIN2A*.

### 3.7 RT‐qPCR Validation

**FIGURE 7:**
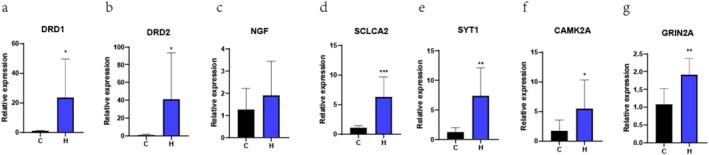
Real‐time quantitative polymerase chain reaction (RT‐qPCR) validation of RNA sequencing (RNA‐seq) data for the differential expression of seven genes (a–g). *N* = 6 in each group. *p < 0.05, **p < 0.01 and ***p < 0.001 compared with the control group.

RT‐qPCR was used to validate data quality. Seven genes, NGF, DRD1, DRD2, SLC1A2, CAMK2A, SYT1 and GRIN2A, were associated with the dysregulation of neurotransmitter systems, dysfunction in calcium signalling and membrane potential regulation. The RT‐qPCR results showed the same expression trend as that of the transcriptome. Moreover, NGF, DRD1, DRD2, SLC1A2, CAMK2A, SYT1 and GRIN2A showed substantial differences (Figure 7). These results are consistent with those observed in the transcriptome analysis, thereby demonstrating the reproducibility of the transcriptome data.

## 4 Discussion

In this study, adult male rats were treated with a wide spectrum of MCAT doses (0.25, 5 and 20 mg/kg)—ranging from subthreshold to severely toxic—to evaluate its impact on learning, memory and synaptic morphology in the PFC. The MWM is a widely used behavioural test in the field of neuroscience for evaluating learning and memory functions in rodents. The MWM test showed that chronic MCAT exposure led to impaired learning and memory in rats.

The PFC mediates learning and memory. Synaptic plasticity is directly related to learning and memory, and dendritic spines are considered the morphological basis of synaptic plasticity [30]. A decrease in the density of dendritic spines leads to decreased synaptic plasticity, which consequently affects learning and memory. TEM showed that MCAT exposure destroyed the structure of synapses in rat prefrontal cortical neurons, including the synaptic cleft and dense postsynaptic layer. Moreover, Golgi staining showed that MCAT exposure decreased dendritic spine density. Therefore, the behavioural and morphological experiments confirmed the successful establishment of a rat model in which chronic MCAT exposure impaired learning, memory and the synaptic structure of prefrontal cortical neurons.

DEGs in the PFC of male rats exposed to high MCAT concentrations were compared with those in a blank control group. This was done to determine the potential mechanism of MCAT‐induced learning and memory dysfunction in rats and to identify possible genes or pathways that facilitated the observed neurobehavioral changes. To gain a deeper understanding of the functions of the DEGs and the main signal transduction and biochemical metabolic pathways involved in their transcriptional proteins, GO enrichment and KEGG pathway analyses were performed. A total of 1457 (694 up‐regulated and 763 down‐regulated) genes were identified in the high‐concentration and blank control groups. KEGG pathway analysis showed that DEGs were significantly enriched in “neuroactive ligand–receptor interaction,” “PI3K–Akt signalling pathway,” “calcium signalling pathway,” “cAMP signalling pathway,” “MAPK signalling pathway,” “Alzheimer disease” and “GABAergic synapse,” especially in “neuroactive ligand–receptor interaction,” where up to 67 DEGs were enriched. GO functional analysis showed that the DEGs were enriched in “regulation of membrane potential,” “channel activity,” “ion channel activity,” “calcium ion transport,” “synaptic membrane,” “signalling receptor activator activity,” “receptor ligand activity,” “regulation of metal ion transport” and “regulation of neurotransmitter levels.” These enrichment results indicate that dysregulation of neurotransmitter systems, calcium signalling and membrane potential regulation affect the structure of synapses and participate in MCAT‐induced neurotoxicity.

### 4.1 Dysregulation of Neurotransmitter Systems

KEGG pathway enrichment analysis showed that 67 DEGs were enriched in “neuroactive ligand–receptor interactions.” Neuroactive ligand–receptor interactions regulate neurotransmitter transduction and synaptic plasticity in neurons, leading to changes in learning and memory functions [31, 32]. GO enrichment analysis was enriched in entries related to neurotransmitter regulation, including “regulation of neurotransmitter levels,” “neurotransmitter transport,” “neurotransmitter secretion,” “regulation of neurotransmitter transport,” “regulation of neurotransmitter secretion,” “neurotransmitter receptor activity” and “postsynaptic neurotransmitter receptor activity.” These included changes in the expression of glutamatergic, GABAergic, cholinergic, DAergic and serotonergic genes. These included GABA receptor A4 (Gabra4), B2 (B2), G3 (Gabrg3), R1 (Gabrr1), subunit theta (Gabrq), A3 (Gabra3) and B3 (Gabrb3) in the GABA system; metabotropic glutamate receptor 6 (Grm6), Grin3a, glutamate receptor, ionotropic, kainate, 3 (Grik3) and Grin2a in the glutamate system; 5‐HT receptor 5B (Htr5b), 1D (Htr1d), 1B (Htr1b) and 6 (Htr6) in the 5‐HT system; cholinergic receptor nicotinic beta 3 (Chrnb3) and 2 (Chrnb2) in the neuronal acetylcholine (Ach) system; Drd1 and Drd2 in the DA system and thyroid stimulating hormone subunit beta (Tshb) and thyrotropin‐releasing hormone (Trh) and receptor (Trhr) in the thyroid hormone system. The PFC is a brain region associated with higher‐order cognitive and behavioural processes. Memory formation is a complex process that involves various neurotransmitter systems. The following three major systems are usually involved and play a major role in this process: glutamatergic, GABAergic and cholinergic systems [33]. Normal brain function depends on a delicate balance between excitatory and inhibitory neurotransmission, mediated mainly by glutamatergic and GABA signalling. Disruption of this balance affects synaptic plasticity, impairs learning and memory and results in several neurological diseases [34, 35, 36]. ACh is a neurotransmitter of the cholinergic system that is released within the hippocampal circuit and is important for learning and memory. Moreover, ACh is a powerful presynaptic regulator of glutamatergic and GABAergic synaptic transmission [37]. Changes in the activity of the 5‐HT and DA neurotransmitters also cause changes in the structural patterns of neuronal cells, which affect the organisation of information related to learning and memory [38, 39, 40, 41]. Thyroid hormone has a variety of physiological effects and is essential for normal behaviour, intelligence and neural development, including synapse formation, growth of dendrites and axons, myelination and neuronal function [42, 43]. Other DEGs including adenosine A2A receptor (Adora2a), oxytocin receptor (Oxtr), proopiomelanocortin (Pomc) and learning and memory functions are also closely related [44, 45, 46].

### 4.2 Dysfunction in Calcium Signalling

Signal transduction is the process by which chemical or physical signals are transmitted through cells through a series of molecular events. This is a fundamental mechanism that controls cell growth, proliferation and metabolism. GO enrichment analysis of DEGs was significantly enriched in “signalling receptor activator activity” and “receptor ligand activity.” Moreover, KEGG annotation indicated that the “PI3K–Akt signalling pathway,” “cAMP signalling pathway,” “calcium signalling pathway” and “MAPK signalling pathway,” which are closely related to neurodegenerative diseases, were involved. Particularly, dysfunction in calcium signalling leads to either excessive or insufficient calcium levels within neurons [47]. The GO entries of differentially expressed mRNAs were enriched in terms related to disruption of calcium homeostasis, including “calcium ion transport,” “calcium ion homeostasis,” “regulation of calcium ion transport,” “cellular calcium ion homeostasis,” “calcium ion transmembrane import into cytosol,” “positive regulation of cytosolic calcium ion concentration,” “release of sequestered calcium ion into cytosol,” “negative regulation of sequestering of calcium ion,” “sequestering of calcium ion,” “regulation of release of sequestered calcium ion into cytosol,” “positive regulation of release of sequestered calcium ion into cytosol” and “voltage‐gated calcium channel activity.” As a ubiquitous second messenger, Ca^2+^ plays an important role in cell signalling and is involved in neuronal functions, such as neuronal transmission, neurogenesis and synaptic plasticity. Ca^2+^ regulation involves multiple signalling processes; however, the ultimate goal is to maintain homeostasis of intracellular Ca^2+^ concentrations [48, 49]. Changes in Ca^2+^ levels have been associated with a variety of diseases, some of which are neurodegenerative, including Alzheimer's disease, psychiatric disorders and metabolic disorders [50, 51]. Ca^2+^ signalling is complex and involves interactions at multiple activation sites, including specialised receptors, Ca^2+^‐binding proteins and ion exchangers on the membranes of the cytoplasm and intracellular organelles (endoplasmic reticulum and mitochondria) [52]. This renders the Ca^2+^ signalling pathway a target for a range of pathological events that disrupt homeostasis of the cellular environment.

### 4.3 Membrane Potential Regulation

Membrane potentials regulate excitatory synapses in the cerebral cortex. Although action potentials (APs) are necessary to initiate information transfer in the mammalian cerebral cortex, experimental animals have demonstrated that presynaptic signals below the AP threshold (i.e., subthreshold signals) modulate synaptic strength [53]. For example, in synapses between pyramidal neurons in the cerebral cortex of ferrets and rats, subthreshold depolarisation preceding APs lasting > 1 s resulted in an increase in synaptic amplitude [53, 54]. Through this mechanism, membrane potential states modulate local synaptic networks to promote long‐term synaptic plasticity, which underlies memory consolidation [55]. MCAT is an illegal stimulant synthesised by the oxidation of ephedrine or pseudoephedrine with potassium permanganate. The neurotoxic effects in MCAT users are attributable to Mn, which has an ionic radius similar to that of Ca^2+^ and acts as a Ca^2+^ analog. Mn^2+^ enters excitable cells through voltage‐gated Ca^2+^ channels and Na^+^/Ca^2+^ exchangers, thereby interfering with cation transport in cells and consequently affecting a wide range of related ion transport pathways and gated channels [56, 57]. Ion channels are important regulators of excitability, network activity and plasticity. Changes in the expression and function of membrane K^+^, Na^+^ and Ca^2+^ channels alter the excitability and firing of GABAergic and glutamatergic neurons, thereby altering the excitation/inhibition (E/I) balance in neural networks. The E/I balance shapes the response properties of neurons and regulates their plasticity, ultimately leading to changes in cognitive abilities [58, 59, 60]. This was confirmed via GO enrichment analysis of the DEGs. Five of the top 10 enriched items are directly related to changes in membrane potential, including “regulation of membrane potential,” “channel activity,” “ion channel activity,” “calcium ion transport” and metal ion transmembrane transporter activity, which are ranked 1st, 2nd, 4th, 5th and 10th in the GO enriched items, respectively. In addition, the pathways in the KEGG‐enriched items were also closely related to membrane potential, including the cAMP and cyclic guanosine monophosphate signalling pathways and the calcium signalling pathway.

Protein interactions among the 1457 DEGs were predicted using STRING. The top hub genes in the DEGs were evaluated using the maximum cluster centrality method with the CytoNCA plug‐in, which was visualised using Cytoscape software. The DEGs involved in the dysregulation of neurotransmitter systems, dysfunction in calcium signalling and membrane potential regulation were intersected with the top hub genes, and seven overlapping hub genes, including *NGF*, *DRD1*, *DRD2*, *SLC1A2*, *CAMK2A*, *SYT1* and *GRIN2A*, were identified.

NGF belongs to the family of neurotrophic factors. NGF activates downstream signalling pathways, such as PI3K–Akt and MAPK, through tropomyosin receptor kinase A, promotes synaptic protein synthesis and increases synaptic plasticity [61, 62]. In addition, NGF regulates the release of neurotransmitters, such as Ach, and affects the efficiency of synaptic transmission. The cholinergic system is also important for attention and memory formation. NGF supports synaptic plasticity in the hippocampus and PFC by maintaining cholinergic neurons [63]. Insufficient NGF levels may impair these functions. Administration of NGF improves memory deficits, whereas NGF inhibition impairs learning ability [64].

Increasing evidence suggests that DA receptors play key roles in learning and memory. DA signalling regulates learning, memory and dendritic spine formation. Associative learning and synaptic plasticity in hippocampal CA3–CA1 require DAD1 receptors (D1R) and DAD2 receptors (D2R) [65, 66]. DR1 and DR2 affect synaptic morphology and functional plasticity through interactions with NMDA receptors (NMDAR), α‐amino‐3‐hydroxy‐5‐methyl‐4‐isoxazolepropionic acid receptors and the Ca^2+^/calcium/calmodulin‐dependent protein kinase II (CaMKII)/cAMP response element‐binding protein signalling pathway [67, 68, 69].

SLC1A2 is mainly expressed in glial cells and is the main mechanism for removing extracellular glutamate from the synaptic cleft, regulating glutamate homeostasis and protecting the brain from glutamate‐induced neuronal hyperexcitation and excitotoxicity [70]. The loss or down‐regulation of SLC1A2 expression leads to the accumulation of glutamate in the synaptic cleft and overstimulation of glutamate receptors, which are associated with an excessive influx of Ca^2+^ into neurons [71]. Regulation of glutamate uptake by astrocytic SLC1A2 affects spatial memory [72].

CAMK2A encodes a subunit of CaMKII. Neuronal CAMK2A is mainly localised in postsynaptic sites and dendritic spines and is involved in the functional and structural forms of neuronal plasticity [73]. When calcium flows through NMDARs, CAMK2A is activated. In turn, CAMK2A binds to NMDAR and maintains its open state, leading to further calcium influx and long‐term potentiation. This results in changes in postsynaptic density and dendritic spine volume, thereby affecting learning and memory functions [74].

SYT1 is a key molecule that regulates synaptic vesicle (SV) exocytosis in a Ca^2+^‐dependent manner. SYT1 contains two Ca^2+^‐binding domains, C2A and C2B, and is one of the major calcium sensors at synapses that promote SV exocytosis and neurotransmitter release in response to Ca^2+^ influx [75]. Interestingly, elevated intracellular calcium levels also enhance the production of amyloid‐beta (Aβ) and Aβ42, thereby suggesting that Ca^2+^ influx may regulate amyloid precursor protein (APP) cleavage by β‐ and γ‐secretases. SYT1 acts as a novel presynaptic calcium‐dependent interacting protein of presenilin 1/γ‐secretase, which regulates APP processing in an activity‐dependent manner, thereby regulating Aβ production/secretion at synapses [76]. Furthermore, SYT1 deficiency may affect the generation and release of glutamate and γ‐aminobutyric acid by regulating the release process of SVs [77].

GRIN2A encodes a member of the glutamate‐gated ion channel protein family. GRIN2A plays an important role not only in synaptic plasticity but also in learning and memory. Furthermore, impairments in spatial or discrimination learning were observed in mice with a knockout of the GRIN2A subunit [78]. Dendritic branch pruning is accompanied by maturation and an increase in GRIN2A levels. GRIN2A loss reduces the total dendritic length and complexity of hippocampal dentate gyrus neurons located in the internal granular zone [79]. Knockdown of astrocytic GRIN2A aggravates Aβ‐induced memory and cognitive deficits by regulating NGFs [80].

In summary, low, medium and high MCAT concentrations cause learning and memory dysfunction and destroy the morphological structure of synapses. In total, 1457 DEGs were identified in the PFC of rats in the high‐concentration group, which were substantially enriched in the regulation of the neurotransmitter system, Ca^2+^ stability, membrane potential regulation and synapses compared with that in the control group. The neurotoxicity caused by MCAT may lead to dysregulation of the neurotransmitter system, thereby destroying Ca^2+^ stability and resulting in abnormal changes in membrane potential. This subsequently regulates a variety of synaptic processes, including synapse formation, ion homeostasis, synaptic transmission and plasticity, through a series of signalling pathways, which ultimately results in learning and memory dysfunction.

This study provides multi‐layered evidence for MCAT‐induced neurotoxicity through integrated behavioural, morphological and transcriptomic analyses; however, several limitations should be noted. First, the MWM test used here primarily evaluates spatial reference memory. Future studies should incorporate assessments of other cognitive domains (e.g., working memory via the Y‐maze and recognition memory via novel object recognition) to obtain a more comprehensive profile of cognitive deficits. Second, the sample size for the morphological and transcriptomic analyses was relatively small. Although we observed highly consistent trends, these findings must be considered preliminary and hypothesis‐generating, warranting validation in larger, independent cohorts. Furthermore, while this study establishes a strong correlation between MCAT exposure and various pathological phenotypes, definitive causal inference awaits future gain‐ and loss‐of‐function experiments. Finally, the signalling pathways identified by functional enrichment analysis represent common response networks to diverse neurotoxic substances, reflecting shared terminal pathophysiological pathways. Future work will focus on identifying potential MCAT‐specific upstream triggers or unique gene regulatory network modules within these common pathways to delineate its precise molecular signature.

## Author Contributions

**Rukui Zhou:** Data curation, Investigation, Methodology, Writing – original draft. **Yingwen Xu:** Software, Visualization. **Chunming Xu:** Formal analysis. **zhe Chen:** Supervision. **Jieping Lv:** Conceptualization, Resources. **Keming Yun:** Funding acquisition, Supervision. **Zhiwen Wei:** Funding acquisition, Resources, Writing – review and editing. All the authors have agreed to be responsible for ensuring the accuracy of the presented data.

## Funding

This work is financially supported by the National Key Research and Development Program of China (No. 2022YFC3300903), National Natural Science Foundation of China (No. 82130056), Shanxi Province Science Foundation (No. 202103021224233), and Chinese Medicine Research Project of Shanxi Provincial Administration of Chinese Medicine (No. 2024ZYYC102).

## Ethics Statement

All animal experiments were approved by the Institutional Animal Care and Use Committee of Shanxi Medical University (2021‐338).

## Consent

The authors have nothing to report.

## Conflicts of Interest

The authors declare no conflicts of interest.

## Data Availability

The project reference genome, mRatBN7.2.108, can be downloaded from https://ftp.ensembl.org/pub/release108/fasta/rattus_norvegicus/dna/Rattus_norvegicus.mRatBN7.2.dna.toplevel.fa.gz. The annotation files can be downloaded from: https://ftp.ensembl.org/pub/release‐108/gtf/rattus_norvegicus/Rattus_norvegicus.mRatBN7.2.108.chr.gtf.gz.
